# Evaluating the relationship between lesion burden and aging among the skeletons of an 18^th^-19^th^ century London cemetery using osteological and radiological analysis

**DOI:** 10.1371/journal.pone.0196448

**Published:** 2018-04-26

**Authors:** Katherine van Schaik, Ronald Eisenberg, Jelena Bekvalac, Frank Rühli

**Affiliations:** 1 Harvard Medical School, Boston, Massachusetts, United States of America; 2 Harvard Department of the Classics, Cambridge, Massachusetts, United States of America; 3 Department of Radiology, Beth Israel Deaconess Medical Center, Harvard Medical School, Boston, Massachusetts, United States of America; 4 Centre for Human Bioarchaeology, Museum of London, London, United Kingdom; 5 Institute of Evolutionary Medicine, University of Zurich, Zurich, Switzerland; Hebrew University, ISRAEL

## Abstract

Study of disease in the past can help illuminate patterns of human health, disease, and aging in the present. As average human life expectancy and incidence of chronic disease have increased in the last century, efforts to understand this epidemiologic shift have led to more investigation of healthy aging. Using osteological and radiological methods of analysis, this study examined 212 mostly nineteenth century adult skeletons from the crypt of St. Bride’s in London, in order to investigate the relationship between age-at-death, sex, and number of lesions observed in bone. Lesions were classified into macro-level categories according to the *Rapid Method for Recording Human Skeletal Data*, and the correlation between age group and number of lesions in each category, as well as the total number of lesions, were analyzed. Correlations between age-at-death and the number and type of lesions were compared across both methods of analysis. A greater total number of lesions and a greater number of types of lesions was observed for the osteologically analyzed data, compared to the radiologically analyzed data. Correlations between age-at-death and specific pathology groups were in general weak, though stronger for the osteologically analyzed data. For each method of analysis, there were statistically significant differences between the total number of lesions and age group, with total number of lesions increasing with age, regardless of method of analysis. Joint and metabolic lesions were the most significant predictors of age-at-death. The correlations between total lesions observed and age-at-death were similar for radiologically and osteologically analyzed data, for the same set of bones. This suggests that, for the bones analyzed, while the number of lesions recorded differed according to method of analysis, the relationship between overall observed lesion burden and age-at-death was similar for both osteological and radiological analysis.

## Introduction

Average life expectancy increased globally during the twentieth century and continues to do so in the twenty-first [1]. By the year 2020, “for the first time in history, people aged 65 years and older in the world will outnumber children aged younger than 5 years” [2]. As life expectancy increases, so does the incidence of chronic disease [3]. Efforts to understand this shift in human life expectancy and increased burden of chronic disease have led to more studies investigating aging and its associated biological mechanisms. These studies, many of which focus on the genetic factors correlated with healthy aging, have identified specific genes associated with both extended longevity and disease-free good health [4–9]. However, these genetic factors are not the sole determinants of healthy aging and work in conjunction with lifestyle and environmental factors to facilitate both increased longevity and the absence or delayed onset of such age-associated pathologies as cancer and cardiovascular diseases [10,11]. Longevity genes appear to make only a small contribution to increased survival up to age 60, after which the relationship between longevity genes and survival appears to be stronger [7,8]. Recent research has focused on study of the “wellderly” (individuals older than the age of 80 without chronic disease and taking no medications), sharpening the distinction between the concepts of healthy aging and longevity [9]. Unlike mere longevity, healthy aging has not been associated with decreased rates of pathogenic genetic variants and instead appears to be associated with protective genetic factors that promote healthy aging [9]. Environmental factors also play a role in healthy aging, and the nature of their relationship to genetic factors is still incompletely understood [10,11].

An important aspect of studies about healthy aging involves the relationship between inflammation, aging, and mortality. Although studies have identified a link between inflammation and aging, the causative and correlative aspects of this link have been more difficult to determine [12,13]. What has been clearly identified, however, is that increases in inflammatory markers are associated with the presence of chronic diseases, such as arthritis, gout, and diabetes, as well as with the presence of acute disease [14]. These increases in inflammatory markers, in turn, are associated with functional decline and mortality [14]. Other studies have examined the genetic components of the body’s ability to produce inflammatory proteins, correlating the presence of these proteins with differences in age-related degenerative change [15]. The importance of one particular inflammatory protein in aging was emphasized by research that demonstrates that its ablation protected mice from age-related changes to the immune system and the neurological system [15]. Further research efforts are focusing on identifying causal relationships between inflammation, disease, and aging.

Investigations of the nature of human aging, and the accumulation of pathological change over time, rely implicitly on comparisons with the past. The increase in human life expectancy over the last century, for example, is framed in comparative terms: on average, humans live longer now than previously in human history. Advances in hygiene, increased understanding of human physiology and genetics, and development and use of technology have facilitated increased longevity and healthy aging. Nevertheless, the presence of genetic components of longevity and healthy aging suggest that in the past there also existed a population of individuals who were healthier and lived to an older age in comparison to their average contemporaries. These individuals could have responded differently to pro-inflammatory states, based on their genotypes. Analysis of such past treatment-naïve “wellderly” populations, who attained greater ages and maintained their health without the modern medical technologies that can affect studies of the wellderly today [9], can provide additional insight into the process of aging.

Study of disease in the past has already contributed to debates about the nature of human aging through projects like the Horus study [16], in which whole-body CT scans were taken of mummies from four different populations and time periods. This study concluded that “although commonly assumed to be a modern disease, the presence of atherosclerosis in premodern human beings raises the possibility of a more basic predisposition to the disease,” lending support to arguments about the role of stress and inflammation in aging [16]. Additional research has identified genomic correlates of cardiovascular disease in the South Tyrolean Iceman, who lived 5,000 years ago [17]. These findings have broadened our understanding of human aging across different time periods and have encouraged reconsideration of arguments about potential disease-causing aspects of ‘the modern lifestyle’ [18–20]. Consequently, the primary goal of this project was to investigate the relationship between disease and age-at-death using data from the past in order to gain a more nuanced appreciation of the relationship between age and disease.

The relationship between age-at-death and disease burden (broadly defined) is difficult to identify precisely in bioarchaeological contexts for many reasons, and DeWitte and Stojanowsky discuss these issues in detail in their extensive literature review [21]. Challenges in identifying the relationship between age-at-death and disease burden that relate most directly to this study include: 1) The methods used to determine age-at-death (in absence of historical records) are of variable accuracy and precision. 2) Study populations are often small, and the potential for generalizability of results is limited. 3) Only pathological conditions that manifest in bone are observable, and these are observable to different degrees with different methods (i.e., osteological analysis or radiological analysis). 4) The principles of “heterogeneity in frailty” (where frailty is defined as the age-standardized relative risk of death) and “selective mortality” affect the interpretation of results. Both principles emphasize the variability in individuals’ risks of death and disease, and the potential for frail individuals to be overrepresented in mortuary samples [21–24]: in other words, by the time these individuals died, there was a greater probability that they had become frail, compared to others in their population.

These four challenges in understanding the relationship between pathology and age-at-death drove this investigation. We sought to quantify, using two different methods, the bone lesions for a sample for which age-at-death was known through historical records, and to determine the relationship between these bone lesions and historically verified age-at-death. Although we were unable to use DNA data from our samples, we instead relied on assumptions drawn from data linking aging and increased mortality with the pro-inflammatory states associated with both acute and chronic disease. Since modern studies show a correlation between inflammation (linked to the presence of acute and chronic disease) and increased mortality, we sought to investigate the potential presence of a correlation between lesions observed in bone and age-at-death. Although most recent paleopathology studies focus on specific frailty indicators that have clear statistical relationships with age-at-death [21], our study takes a global approach, considering the relationship between age-at-death and the number of lesions observed in macro-level categories. Our justification for making this globalized comparison rests in correlations that have been demonstrated between inflammation, chronic disease, aging, and mortality [12,13]: we therefore took into account multiple potential sources of pathology in examining the relationship between lesion accumulation and age, including those that might not seem to have immediate bearing on cause of death (i.e., congenital factors). We had two goals in carrying out this study: first, to analyze the correlation between age-at-death and the number of lesions observed, using two different methods of analysis, and second, to characterize potential differences in lesion identification using osteological and radiological analysis.

## Materials and methods

One particular subset of the collection of human remains at St. Bride’s Church in London, in the curatorial care of the Museum of London’s Centre for Human Bioarchaeology, provided an appropriate study population to investigate the relationship between bone lesions and age-at-death. This subset is discussed below, in the context of additional background information about the individuals interred at St. Bride’s.

The study population we used consists of 227 skeletons (213 individuals aged 18 or older at the time of their death, and 14 sub-adults) from the eighteenth and nineteenth centuries (S1 Table). Historically verifiable identities and age-at-death data are available from the name plates attached to the coffins in which the skeletons were found when the church was excavated and rebuilt following bombing during the Second World War [25]. Because our study is a methodological comparison, with particular focus on questions related to lesion burden and age-at-death, we used a study population for which historically verifiable age-at-death data are available. Although, as described in Milne’s Archaeological Survey of St. Bride’s [25], thousands of individuals have been buried on this site since the medieval period, we only studied those from the 18^th^ and 19^th^ century collection for which lead coffin plates are available. This collection has been cataloged and examined before [26], though without systematic radiographic analysis.

In this collection of 227 individuals from the 18^th^ and 19^th^ centuries for which coffin plates are available, the skeletons are well-preserved, with 88% more than 50% complete and 73% more than 60% complete (with percent completeness referring to the presence or absence of a bone, provided that 50% or more of that bone remained). Adult skeletons were evaluated for evidence of lesions by an osteologist, and digital radiographs of the crania, humeri, femora, tibiae, and pelvises were evaluated for evidence of lesions by a radiologist. Sub-adults were excluded from the study, as they represented a range of ages and therefore had bones in various stages of development. Definitive reasons for the precise ratio of adults to sub-adults (213 adults, 14 sub-adults) interred in coffins with lead coffin plates during the 18^th^ and 19^th^ centuries have not been extensively characterized but may be related to the burial practices of the time [26]. We limited our study to analysis of only adult skeletons to facilitate consistency of the study sample. Furthermore, one individual of the 213 adults in the total collection was excluded from the statistical analysis because damage to the associated coffin plate prevented accurate determination of his age-at-death. The N for the purpose of statistical analysis was therefore 212.

No permits were required for this study, which complied with all relevant regulations. The skeletons were examined by the same experienced osteologist, who noted and recorded all lesions in accordance with the Museum of London’s *Human Osteology Method Statement* [27], following the procedure outlined in the *Rapid Method for Recording Human Skeletal Data* [27,28]. The skeletons are held in the crypt of St. Bride’s Church, London, under the care of the Museum of London, in a permanent repository. They are accessible with permission from the Museum of London. Context numbers are available from the Museum of London’s website.

Age-at-death, sex, and evidence of lesions were recorded in the Wellcome Osteological Research Database (WORD) in accordance with the standard recording protocol. These lesions were classified into macro-level pathology categories as outlined in the *Rapid Method* manual (congenital, infectious, joints, trauma, metabolic, endocrine, neoplastic, circulatory, and miscellaneous), and these macro-levels were further differentiated into more specific groups (see S2 Table). When possible, degree of severity was recorded as a quantifiable number based on accepted criteria (e.g., for cribra orbitalia, vertebral facets, presence of osteophytes, and certain dental pathologies). The number of lesions observed in each of the macro-level pathology categories was recorded for each individual (S3 Table).

Radiographic imaging was carried out on crania (excluding dental pathology), femora, tibiae, pelvises (including the sacrum), and humeri with a Sedecal 4.0 kW X-Ray generator and a Canon Lanmix 35cm x 43cm flat plate digital detector. Cranial radiographs and photographs had been previously obtained in 2010–11 for a separate project, using the same radiographic equipment and radiographer involved in the present study. Radiographs and photographs of the postcranial skeleton were taken specifically for this study. Analysis of the radiographs was completed by an experienced radiologist using a DICOM viewer. When initially viewing the images for the presence of lesions, the evaluating radiologist only knew the individual’s sex and age-at-death. Multiple photographs of each bone were available in case questions arose regarding whether a finding was evidence of a lesion or postmortem damage. Lesions observed were classified into the same groups as those given in the *Rapid Method* manual to ensure consistency of data recording for the statistical analysis, and the number of lesions observed in each of the macro-level pathology categories was recorded for each individual.

## Results

All data used in this study are publicly available on the website of the Centre for Human Bioarcheology (Museum of London). Three data sets were used for statistical analysis. The first set, called the UOD (unadjusted osteological data), included all data recorded from all bones. The second set, called the RD (radiological data) consisted of data acquired from crania, humeri, pelvises, femora, and tibiae. The third set, called the AOD (adjusted osteological data), consisted of the observations made by the osteologist for only those bones included in the radiological analysis. This adjusted data set allowed for matched comparison of the two methods of analysis. Tables 1, 2 and 3 show the average number of lesions of each type for each age group, for all 3 data sets.

**Table 1:** Average number of lesions per age group for each pathology group, UOD.

Age Group (n)	Congenital	Infectious	Joint	Trauma	Metabolic	Endocrine	Neoplastic	Circulatory	Miscellaneous	Cribra Orbitalia	Total
**18–25 (19)**	.26	.42	.16	.11	0	0	0	.11	1.26	.47	**2.79**
**26–35 (26)**	.46	.62	.19	.27	.15	0	.04	.08	1.19	.08	**3.08**
**36–45 (28)**	.75	.54	.43	.46	.11	0	.07	.07	1.39	.04	**3.86**
**46–55 (23)**	.7	.48	.35	.13	.04	0	0	.09	1.65	.09	**3.53**
**56–65 (57)**	.65	.53	.79	.4	.05	0	.16	.14	1.47	.18	**4.37**
**66–75 (35)**	.6	.57	1.51	.51	.17	0	.2	.03	1.57	.14	**5.30**
**76+ (24)**	.67	.5	1.25	.54	.33	0	.13	0	1.5	0	**4.92**

**Table 2:** Average number of lesions per age group for each pathology group, RD.

Age Group (n)	Congenital	Infectious	Joint	Trauma	Metabolic	Endocrine	Neoplastic	Circulatory	Miscellaneous	Cribra Orbitalia	Total
**18–25 (19)**	.05	.11	0	.05	.11	0	0	0	.26	0	**0.58**
**26–35 (26)**	.04	.15	.08	.12	.15	0	0	.08	.12	0	**0.74**
**36–45 (28)**	.04	.07	.18	.04	.18	0	0	0	.14	0	**0.65**
**46–55 (23)**	.13	.04	.43	0	.13	0	.09	0	.22	0	**1.04**
**56–65 (57)**	.02	.21	.54	.02	.39	0	.09	.04	.49	0	**1.80**
**66–75 (35)**	.09	.06	.51	.14	.29	0	.11	0	.11	0	**1.31**
**76+ (24)**	0	.04	.58	.13	.25	0	.04	0	.29	0	**1.33**

**Table 3:** Average number of lesions per age group for each pathology group, AOD.

Age Group (n)	Congenital	Infectious	Joint	Trauma	Metabolic	Endocrine	Neoplastic	Circulatory	Miscellaneous	Cribra Orbitalia	Total
**18–25 (19)**	.11	.42	.11	.05	0	0	0	0	.47	.47	**1.63**
**26–35 (26)**	.19	.46	.15	.19	.04	0	.04	.08	.54	.08	**1.77**
**36–45 (28)**	.39	.39	.07	.25	.11	0	.07	.04	.79	.04	**2.15**
**46–55 (23)**	.3	.35	.13	.04	0	0	0	0	.87	.09	**1.78**
**56–65 (57)**	.3	.44	.25	.14	.05	0	.14	.05	.91	.18	**2.46**
**66–75 (35)**	.23	.51	.46	.23	.17	0	.2	0	1.03	.14	**2.97**
**76+ (24)**	.33	.5	.46	.21	.33	0	.13	0	1.04	0	**3**

The data do not suggest a direct correlation between total number of lesions and age at death, although across all data sets and both methods of analysis, there is a statistically significant difference between the age groups’ mean total number of lesions (UOD: df = 6, F = 4.758, p <0.01; AOD: df = 6, F = 3.474, p <0.01; RD: df = 6, F = 5.932, p <0.01). In other words, as age group increases, there is a statistically significant increase in the total number of observed lesions. An ordered Tukey’s test comparing each age group with every other age group, for all three data sets, did not provide evidence that older age groups have less total lesions for all datasets. There was no significant difference (at the 0.05 level) between sex and mean number of lesions for any data set (UOD: t = 1.01, df = 209.99, p-value = 0.31; AOD: t = -0.76, df = 201.04, p-value = 0.45; RD: t = -1.70, df = 209.39, p-value = 0.09).

**Fig 1:**
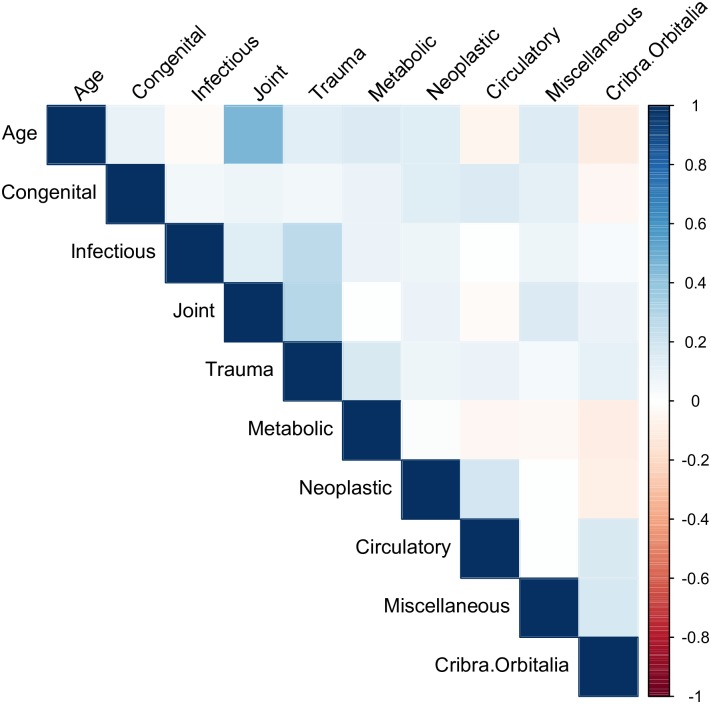
Correlation Matrix for unadjusted osteological data.

**Fig 2:**
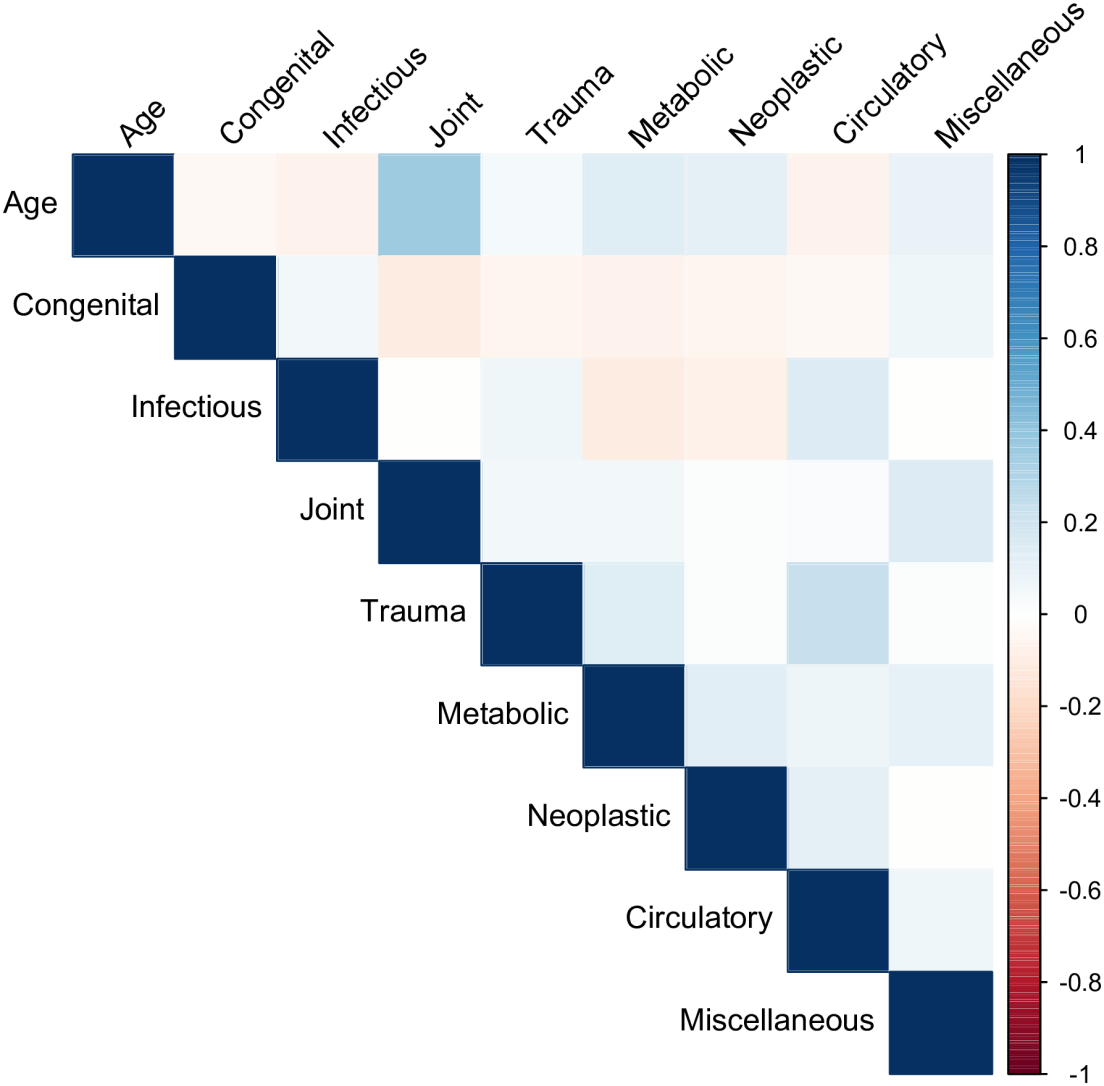
Correlation Matrix for radiological data.

**Fig 3:**
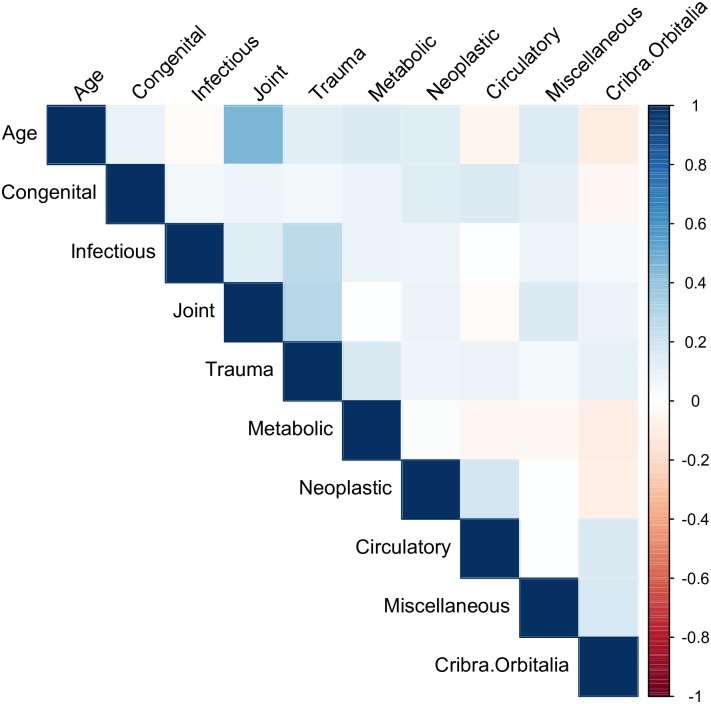
Correlation Matrix for adjusted osteological data.

The correlations between total number of lesions and age-at-death were weak to moderate and fairly similar for all three datasets (UOD = 0.31, AOD = 0.27, RD = 0.28). Figs 1–3 show correlation matrices between age-at-death and pathology group. In general, the correlations are weak to moderate: the strongest correlation is between joints and age-at-death for the UOD (0.46), followed by joints and age-at-death for the RD (0.35).

In an effort to develop a model to predict age-at-death based on sex and lesion burden, regression analysis was undertaken. A regression to predict age-at-death given the type of pathology for the UOD found that, among the variables included in the regression, sex was not a significant predictor of age-at-death. Consequently, the regression model chosen included the number of lesions in each pathology category, across all categories. This model found the number of joint lesions to be the most significant predictor of age-at-death (p <0.01), followed by the number of lesions in the metabolic category (p = 0.02), and the number of lesions representative of cribra orbitalia (measured as no evidence of cribra orbitalia, evidence in one orbit, or evidence in both orbits, p = 0.05). For the AOD, sex was similarly not a significant predictor of age-at-death and so was excluded as a variable in the regression analysis. The most significant predictors in this model were the number of lesions in the miscellaneous group (p < 0.01), followed by the number of lesions in the joints (p < 0.01) and metabolic (p < 0.01) categories. For the RD, sex was similarly not a predictor of age-at-death. The number of joint lesions was the most significant predictor (p < 0.01).

The correlation between the total number of lesions recorded and percent completeness of the skeleton (calculated only for the UOD) was 0.46.

## Conclusions

In both historical and modern contexts, the relationships among age, longevity, health, and signs of disease or ill-health are difficult to characterize. Our findings indicated that, for our sample, the correlation between age-at-death and total observed lesions remained fairly constant, regardless of method of analysis. Moreover, this correlation remained similar even when the osteologically-analyzed data were adjusted to include findings only in the same bones that were used for radiological analysis. The correlation between total lesions recorded for the unadjusted osteologically-analyzed data and the percent completeness of the skeleton (0.46) suggests that the total number of recorded lesions is affected by percentage completeness. However, the similarity of the correlation between total number of lesions and age-at-death for the UOD and AOD implies that, at least for the metrics and bones used in this study, limiting the bones included in the analysis may not have substantially altered the overall picture of the correlation between total observed lesions and age.

Resolution of the total observed lesions into more specific categories reveals differences in the correlation between age-at-death and the lesions that most strongly predict age. As shown in both this and previous studies [29,30], joint lesions (as evidence of ‘wear-and-tear’) can be a strong predictor of age-at-death, regardless of method of analysis.

Paleopathological projects cannot yet approach questions about the genetics and biomolecular dimensions of aging with the same degree of precision as can studies of modern, living humans [21]. However, investigation of disease in the past can encourage us to question our ideas about causality, as in the Horus study [16]. Similarly, this study sought to investigate our notions about health and longevity in the past. Analysis revealed statistically significant differences between age groups and total number of lesions, with increasing age group correlating with increasing lesion burden. Although the >75 age group had less total lesions than the 66–75 age group for the UOD, this difference was not statistically significant. In the radiologically analyzed sample, the total number of lesions was nearly the same for the 66–75 age group and the >75 age group. There was no appreciable increase in the number of lesions as age increased.

The comparisons between methods of analysis raise additional questions about the lesion types that are identified, and in what contexts. It is notable that the correlation between total lesions observed and age-at-death were similar for the RD and the AOD. This suggests that, at least for the cranium, humeri, pelvis, femora, and tibiae, the relationship between overall observed lesion burden and age is similar for both methods of analysis. Since there were differences in the total number of lesions measured for the RD and the AOD, future analysis might focus more specifically on particular bones and the kinds of lesions identified using osteological and radiological methods. In modern medical practice, the diagnostic methods and interpretive frameworks that one uses affect what is considered normal or abnormal, and often multiple diagnostic modalities (e.g., imaging and lab tests) can yield more precise information about a diagnosis. Similarly, when searching for evidence of disease in past populations, the use of two methods of analysis can provide a potential counterbalance for ‘over-calling’ or ‘under-calling’ the presence of a lesion in bone.

Though our study was able to address four sources of uncertainty in the interpretation of heterogeneity in frailty (identifying age-at-death and sex through historical records, ensuring relative consistency of source community though focusing on one site, and using a two-fold method of identification and analysis of bone lesions), other sources of uncertainty remain. These include: paucity of additional information regarding the individuals studied (cause of death and individual exposure to environmental stressors), extensive timespan over which the individuals in the study lived and died (100+ years), lack of distinction between potentially fatal conditions and generalized degenerative conditions, difficulties in differential diagnosis, and challenges in assessing severity of pathological conditions from the skeletal remains. Such limitations caution against over-interpreting the results; however, our findings are consistent with research about heterogeneity in frailty that, while not controlling for sex and age-at-death with historical records, control for other interpretive challenges (such as cause of death and timespan over which interment occurred) [30].

In conclusion, our study concurs with previous research emphasizing the predictive value of joint lesions as indicative of age-at-death, and builds on earlier studies by showing that this trend is observed when both osteological and radiological analysis are used. Our results provide evidence that, for specific bones, radiological and osteological analysis can yield broadly similar pictures of the overall burden of disease. Finally, while our study does not show statistically significant evidence of a ‘wellderly’ population (as measured by statistically significant decreases in the lesion burden, below what might be expected in comparison to the other age groups), the data do suggest a possible leveling off of lesion burden above a particular age, though this suggested interpretation requires further analysis.

## Supporting information

S1 Table Specimen numbers for St. Bride’s skeletons used in this study. (DOCX)

S2 Table Macro-level groups and sub-groups (as Adapted from the Museum of London’s *Rapid Recording Manual*). (DOCX)

S3 Table St. Bride’s pathology data. (XLSX)
