# Supplementary material for: Evaluating the relationship between lesion burden and aging among the skeletons of an 18th-19th century London cemetery using osteological and radiological analysis
Source: PLoS One. 2018 Apr 26;13(4):e0196448. doi: 10.1371/journal.pone.0196448 (PMC5919625; doi:10.1371/journal.pone.0196448)
Supplement: S1 Table — (DOCX) [file pone.0196448.s001.docx]

S1 Table. Specimen Numbers for St. Bride’s Skeletons Used in this Study

| Sitecode | Context |
| --- | --- |
| SB79 | 1 |
| SB79 | 2 |
| SB79 | 3 |
| SB79 | 4 |
| SB79 | 5 |
| SB79 | 6 |
| SB79 | 7 |
| SB79 | 8 |
| SB79 | 9 |
| SB79 | 10 |
| SB79 | 11 |
| SB79 | 12 |
| SB79 | 13 |
| SB79 | 14 |
| SB79 | 15 |
| SB79 | 16 |
| SB79 | 17 |
| SB79 | 18 |
| SB79 | 20 |
| SB79 | 21 |
| SB79 | 22 |
| SB79 | 26 |
| SB79 | 27 |
| SB79 | 28 |
| SB79 | 29 |
| SB79 | 31 |
| SB79 | 33 |
| SB79 | 42 |
| SB79 | 43 |
| SB79 | 44 |
| SB79 | 45 |
| SB79 | 46 |
| SB79 | 47 |
| SB79 | 48 |
| SB79 | 49 |
| SB79 | 50 |
| SB79 | 51 |
| SB79 | 52 |
| SB79 | 53 |
| SB79 | 54 |
| SB79 | 55 |
| SB79 | 56 |
| SB79 | 57 |
| SB79 | 58 |
| SB79 | 59 |
| SB79 | 60 |
| SB79 | 61 |
| SB79 | 62 |
| SB79 | 63 |
| SB79 | 64 |
| SB79 | 65 |
| SB79 | 66 |
| SB79 | 67 |
| SB79 | 68 |
| SB79 | 69 |
| SB79 | 70 |
| SB79 | 71 |
| SB79 | 72 |
| SB79 | 73 |
| SB79 | 74 |
| SB79 | 75 |
| SB79 | 76 |
| SB79 | 77 |
| SB79 | 78 |
| SB79 | 79 |
| SB79 | 80 |
| SB79 | 81 |
| SB79 | 82 |
| SB79 | 83 |
| SB79 | 84 |
| SB79 | 85 |
| SB79 | 86 |
| SB79 | 87 |
| SB79 | 88 |
| SB79 | 89 |
| SB79 | 90 |
| SB79 | 91 |
| SB79 | 92 |
| SB79 | 93 |
| SB79 | 94 |
| SB79 | 95 |
| SB79 | 96 |
| SB79 | 97 |
| SB79 | 98 |
| SB79 | 99 |
| SB79 | 100 |
| SB79 | 101 |
| SB79 | 102 |
| SB79 | 103 |
| SB79 | 104 |
| SB79 | 105 |
| SB79 | 106 |
| SB79 | 107 |
| SB79 | 108 |
| SB79 | 109 |
| SB79 | 110 |
| SB79 | 111 |
| SB79 | 112 |
| SB79 | 113 |
| SB79 | 114 |
| SB79 | 115 |
| SB79 | 116 |
| SB79 | 117 |
| SB79 | 118 |
| SB79 | 119 |
| SB79 | 120 |
| SB79 | 121 |
| SB79 | 122 |
| SB79 | 123 |
| SB79 | 124 |
| SB79 | 125 |
| SB79 | 126 |
| SB79 | 127 |
| SB79 | 128 |
| SB79 | 129 |
| SB79 | 130 |
| SB79 | 131 |
| SB79 | 132 |
| SB79 | 133 |
| SB79 | 134 |
| SB79 | 135 |
| SB79 | 136 |
| SB79 | 137 |
| SB79 | 138 |
| SB79 | 139 |
| SB79 | 140 |
| SB79 | 141 |
| SB79 | 143 |
| SB79 | 144 |
| SB79 | 145 |
| SB79 | 146 |
| SB79 | 147 |
| SB79 | 148 |
| SB79 | 149 |
| SB79 | 150 |
| SB79 | 151 |
| SB79 | 152 |
| SB79 | 153 |
| SB79 | 154 |
| SB79 | 155 |
| SB79 | 156 |
| SB79 | 157 |
| SB79 | 158 |
| SB79 | 159 |
| SB79 | 160 |
| SB79 | 161 |
| SB79 | 162 |
| SB79 | 163 |
| SB79 | 164 |
| SB79 | 165 |
| SB79 | 166 |
| SB79 | 167 |
| SB79 | 168 |
| SB79 | 169 |
| SB79 | 170 |
| SB79 | 171 |
| SB79 | 172 |
| SB79 | 173 |
| SB79 | 174 |
| SB79 | 175 |
| SB79 | 176 |
| SB79 | 177 |
| SB79 | 178 |
| SB79 | 179 |
| SB79 | 180 |
| SB79 | 181 |
| SB79 | 182 |
| SB79 | 183 |
| SB79 | 184 |
| SB79 | 185 |
| SB79 | 186 |
| SB79 | 187 |
| SB79 | 188 |
| SB79 | 190 |
| SB79 | 191 |
| SB79 | 192 |
| SB79 | 193 |
| SB79 | 194 |
| SB79 | 195 |
| SB79 | 196 |
| SB79 | 197 |
| SB79 | 198 |
| SB79 | 199 |
| SB79 | 200 |
| SB79 | 201 |
| SB79 | 202 |
| SB79 | 203 |
| SB79 | 204 |
| SB79 | 205 |
| SB79 | 206 |
| SB79 | 207 |
| SB79 | 208 |
| SB79 | 209 |
| SB79 | 210 |
| SB79 | 211 |
| SB79 | 212 |
| SB79 | 213 |
| SB79 | 214 |
| SB79 | 215 |
| SB79 | 216 |
| SB79 | 218 |
| SB79 | 219 |
| SB79 | 220 |
| SB79 | 221 |
| SB79 | 222 |
| SB79 | 223 |
| SB79 | 224 |
| SB79 | 225 |
| SB79 | 226 |
| SB79 | 227 |
| SB79 | 228 |
| SB79 | 229 |
| SB79 | 230 |
| SB79 | 231 |
| SB79 | 232 |
| SB79 | 233 |
| SB79 | 234 |
| SB79 | 235 |
| SB79 | 236 |
| SB79 | 237 |
| SB79 | 238 |
| SB79 | 239 |
| SB79 | 240 |
| SB79 | 241 |
| SB79 | 242 |
| SB79 | 243 |
| SB79 | 244 |
