# Supplementary material for: Evaluating the relationship between lesion burden and aging among the skeletons of an 18th-19th century London cemetery using osteological and radiological analysis
Source: PLoS One. 2018 Apr 26;13(4):e0196448. doi: 10.1371/journal.pone.0196448 (PMC5919625; doi:10.1371/journal.pone.0196448)
Supplement: S2 Table — (DOCX) [file pone.0196448.s002.docx]

S2 Table. Macro-Level Groups and Sub-Groups (as Adapted from the Museum of London’s *Rapid Recording Manual*)

| **Macro-Level Group** | **Disease** | **Pathology** |
| --- | --- | --- |
| Congenital | Other congenital | Skull malformation |
|  | Other congenital | Spinal disorders |
|  | Other congenital | Limb abnormality |
|  | Skull malformation | Skull malformation (general) |
|  |  | Anencephaly |
|  |  | Microcephaly |
|  |  | Hydrocephalus |
|  |  | Bathrocephaly |
|  |  | Cleft palate |
|  |  | Craniofacial abnormality |
|  |  | Artificial cranial abnormality |
|  | Spinal disorders | Scoliosis |
|  |  | Kyphosis |
|  |  | Kyphoscoliosis |
|  |  | Spondylolysis (bilateral) |
|  |  | Spondylolysis (unilateral L) |
|  |  | Spondylolysis (unilateral R) |
|  |  | Spondylolisthesis |
|  | Limb abnormality | Achondroplasia |
|  |  | Suspected hip dysplasia if acetabulum missing |
|  |  | Upper limb aplasia/hypoplasia/malformation |
|  |  | Lower limb aplasia/hypoplasia/malformation |
|  |  | DDH |
|  |  | CDH |
|  |  | Congenital talipes equinovarus (CTEV) |
|  |  |  |
|  |  | Other (congenital) |
| Infectious disease codes | Other infectious | Non-specific infection |
|  |  | Specific infection |
|  |  | Miscellaneous-other infections |
|  | Non-specific infection | Non-specific periostitis |
|  |  | Non-specific osteomyelitis |
|  |  | Sclerosing osteomyelitis (Garre) |
|  |  | Non-specific osteitis |
|  | Specific infection | Tuberculosis (*M. tuberculosis/bovis*) |
|  |  | Treponematosis (*Treponema sp.*) |
|  |  | Leprosy (*M. leprae*) |
|  |  | Brucellosis (*Brucella sp.*) |
|  |  | Tuberculosis/brucellosis |
|  |  | Actinomycosis |
|  |  | Typhoid fever |
|  |  | Nocardiosis |
|  |  | Smallpox |
|  |  | Rubella |
|  | Other infection | Mycotic infection (unclassified) |
|  |  | Poliomyelitis |
|  |  | Septic arthropathy (non-specific) |
|  |  | Parasitic infection (unclassified) |
|  |  |  |
| Joints | Other joints | Osteoarthritis |
|  |  | Seronegative spondyloarthropathy |
|  |  | Erosive arthropathy |
|  |  | Joints miscellaneous |
|  | osteoarthritis | Osteoarthritis |
|  |  | Inflammatory osteoarthritis |
|  | Seronegative spondylarthropathy | Ankylosing spondylitis |
|  |  | Reiter’s disease |
|  |  | Psoriatic arthropathy |
|  | Erosive arthropathy | Rheumatoid arthritis (including juvenile forms) |
|  |  | Gout |
|  |  | Enteropathic arthropathy |
|  | Other | Diffuse idiopathic skeletal hyperostosis (DISH) |
|  |  | Rotator cuff disease |
|  |  | Ankyloses |
|  |  | Neurotrophic arthropathy (Charcot joint) |
|  |  | Ochronotic arthropathy |
|  |  | Haemochromatosis |
|  |  | CPPD |
|  |  | Sarcoidosis |
|  |  | Amyloidosis |
|  |  | Other |
|  |  |  |
| Trauma | Other trauma | Surgical intervention |
|  |  | Accidental |
|  |  | Interpersonal violence |
|  | Surgical intervention | Trepanation |
|  |  | Amputation |
|  | Accidental | Healed fracture |
|  |  | Unhealed fracture (atrophic non-union) |
|  |  | Unhealed fracture (hypertrophic non-union) |
|  |  | Fracture separation of epiphysis |
|  |  | Subluxation (full or partial) |
|  |  | Avulsion injury |
|  |  | Compression fracture (vertebrae) |
|  |  | Soft tissue trauma (ossified haematoma/myositis ossificans) |
|  |  | Fracture with secondary infection |
|  |  | Pathological fracture |
|  | Interpersonal violence | Sharp force trauma (edged instrument) healed |
|  |  | Sharp force trauma (edged implement) unhealed |
|  |  | Blunt force trauma (including depressed cranial fracture) healed |
|  |  | Blunt force trauma (including depressed cranial fracture) unhealed |
|  |  | Projectile injury |
|  | Other | Other |
|  |  |  |
| Metabolic | Other metabolic | General |
|  |  | Osteoporosis |
|  | Vitamin D deficiency | Rickets |
|  |  | Osteomalacia |
|  | Vitamin C deficiency | Scurvy |
|  |  |  |
| Endocrine | Pituitary | Pituitary |
|  | Parathyroid | Hyperparathyroidism |
|  |  | Hypoparathyroidism |
|  | Thyroid | Hyperthryoidism |
|  |  | Hypothyroidism |
|  | Other | Other |
|  |  |  |
| Neoplastic | Bone tissue | Osteoma (including button osteoma) |
|  |  | Osteoid osteoma |
|  |  | Osteoblastoma |
|  |  | Osteosarcoma |
|  | Cartilage tissue | Chondroma |
|  |  | Chondroblastoma |
|  |  | Osteochondroma |
|  |  | Chondrosarcoma |
|  | Fibrous connective tissue | FDC (fibrous cortical defect) |
|  |  | Other fibrous tissue (unclassified) |
|  | Neoplastic general | Giant cell reparative granuloma |
|  |  | Meningioma |
|  |  | Osteoclastoma |
|  |  | Ewing’s sarcoma |
|  |  | Bone cysts |
|  | Other | Other (unclassified) |
|  |  |  |
| Circulatory | Osteochondroses | Perthes disease |
|  |  | Osgood-Shlatter’s disease |
|  |  | Sinding-Larsen’s disease |
|  |  | Blount’s disease |
|  |  | Sever’s disease |
|  |  | Kohler’s disease |
|  |  | Freiberg’s disease |
|  |  | Scheuermann’s disease |
|  |  | Calves disease |
|  |  | Keinbock’s disease |
|  |  | Preiser’s disease |
|  | Osteonecroses | Femoral head necrosis |
|  |  | Femoral condyle necrosis |
|  |  | Slipped femoral epiphysis |
|  |  | Idiopathic osteonecrosis |
|  | Osteoarthropathy | Hypertrophic osteoarthropathy |
|  |  |  |
| Miscellaneous | General pathology comments | Any general comments that do not fit in any specific pathology codes |
|  | Blood disorders | Cribra orbitalia left orbit |
|  |  | Cribra orbitalia right orbit |
|  |  | Porotic hyperostosis |
|  |  | Multiple myeloma |
|  |  | Histiocytosis-X |
|  | Dysplasias | Spondyloepiphyseal dysplasia |
|  |  | Diaphyseal aclasis |
|  |  | Dyschondroplasia |
|  |  | Osteopetrosis |
|  |  | Infantile cortical hyperostisis |
|  |  | Diaphyseal dysplasia |
|  |  | Osteogenesis imperfecta |
|  |  | Chromosomal dysplasia |
|  |  | Dysplasia (unclassified) |
|  | Miscellaneous | Paget’s disease |
|  |  | Leontiasis ossea |
|  |  | Hyperostosis frontalis interna |
|  |  | Neuromechanical condition |
|  |  | Endocranial lesions |
|  |  | Dental |
